# P-1801. Optimizing Antimicrobial Therapy in Gram-Negative Bloodstream Infections: Insights from a Pharmacist-Driven Intervention Leveraging Molecular Rapid Diagnostic Testing

**DOI:** 10.1093/ofid/ofae631.1964

**Published:** 2025-01-29

**Authors:** Kaylee E Caniff, Ashlan Kunz Coyne, Chloe Judd, Carolina Orzol, Mohammed Al Musawa, Callan Bleick, Marco R Scipione, Michael J Rybak

**Affiliations:** Anti-Infective Research Lab, Eugene Applebaum College of Pharmacy and Health Sciences, Wayne State University, Royal Oak, Michigan; University of Kentucky College of Pharmacy, Lexington, Kentucky; Wayne State University, Detroit, Michigan; Anti-Infective Research Laboratory, College of Pharmacy and Health Sciences, Wayne State University, Detroit, Michigan; Wayne State University, Detroit, Michigan; Anti-Infective Research Laboratory, Eugene Applebaum College of Pharmacy and Health Sciences, Wayne State University - Detroit (United States); Wayne State University School of Medicine, Department of Microbiology and Immunology - Detroit (United States), Detroit, Michigan; Detroit Receiving Hospital, Detroit, Michigan; Eugene Applebaum College of Pharmacy and Health Sciences, Detroit, Michigan

## Abstract

**Background:**

Molecular rapid diagnostic tests (mRDT) expedite pathogen and antimicrobial resistance gene detection in bloodstream infections (BSI), facilitating the prompt initiation of targeted therapy. However, mRDT has the greatest benefit to patients and public health when integrated with antimicrobial stewardship. This study describes a pharmacist-driven mRDT antimicrobial stewardship intervention in Gram-negative BSI, its associated outcomes and its applicability to public health partners.

**Table 1.**
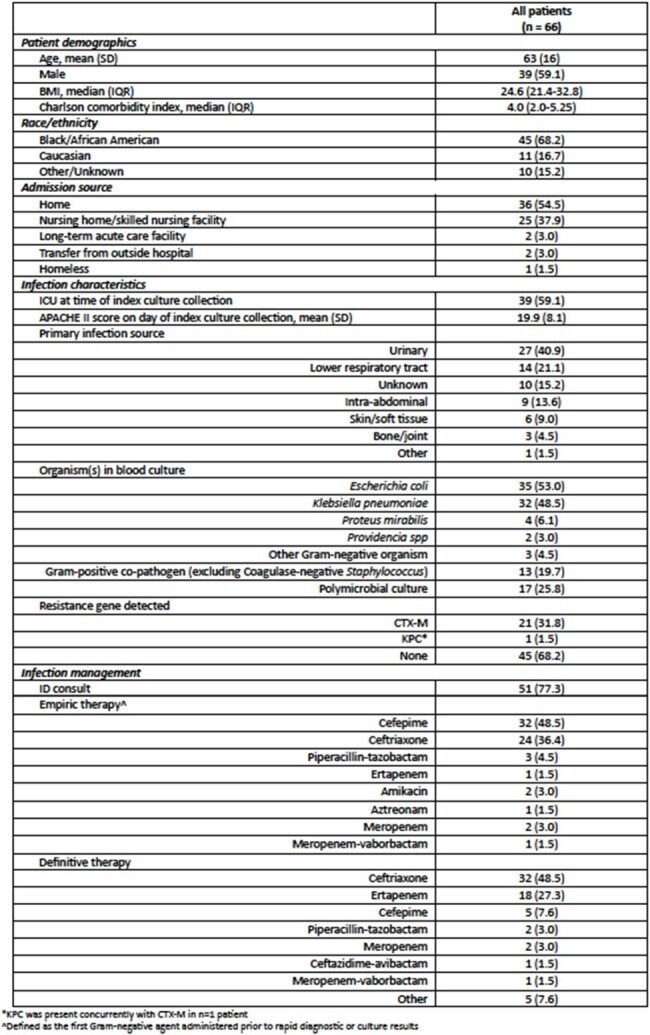
Patient demographics, infection and management characteristics.

**Methods:**

At an urban medical center in Detroit, Michigan, USA, trained pharmacists received an alert page for positive mRDT results. Subsequently, they conducted chart review and provided antimicrobial recommendations to the medical team per site protocol. This is a retrospective, descriptive analysis of patients ≥ 18 years old with Gram-negative BSI who received a pharmacist intervention from 5/2023-4/2024. Patient characteristics and intervention outcomes, including time to pharmacist review and recommendation type, are described.

**Table 2.**
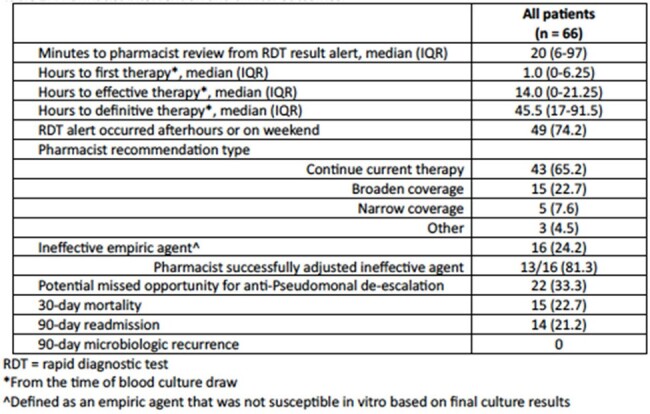
Pharmacist intervention and clinical outcomes.

**Results:**

Sixty-six patients were included; mean (SD) age was 63 (16) years and patients were predominantly Black (68.2%). The most common primary infection source was urinary (40.9%). *Escherichia coli* and *Klebsiella pneumoniae* were most frequently identified in blood culture; 31.8% of isolates possessed CTX-M. Pharmacists reviewed mRDT results at a median (IQR) of 20 (6-97) minutes post-alert. There was no difference in time to effective therapy when mRDT alerts occurred afterhours vs. daytime hours (9 [IQR 0-22] vs. 17 [IQR 0-20] hours, p = 0.66). Sixteen patients were on ineffective empiric therapy; a pharmacist successfully adjusted therapy in 13 of these patients; two patients did not have a resistance marker identified on the initial mRDT report and the pharmacist recommendation was rejected in one patient. Twenty-two patients received empiric anti-Pseudomonal therapy who could have potentially been de-escalated upon mRDT result.

**Figure 1.**
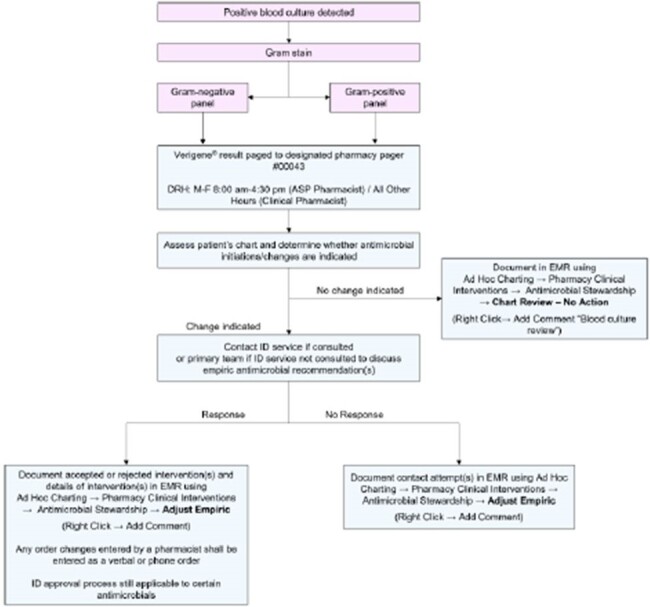
Pharmacist workflow for mRDT alert.

**Conclusion:**

Pharmacists facilitated the attainment of effective therapy in those with Gram-negative BSI with mRDT resistance marker detection. Opportunities for de-escalation highlight the potential for pharmacists to further impact patient care and public health broadly through antimicrobial stewardship.

**Figure 2.**
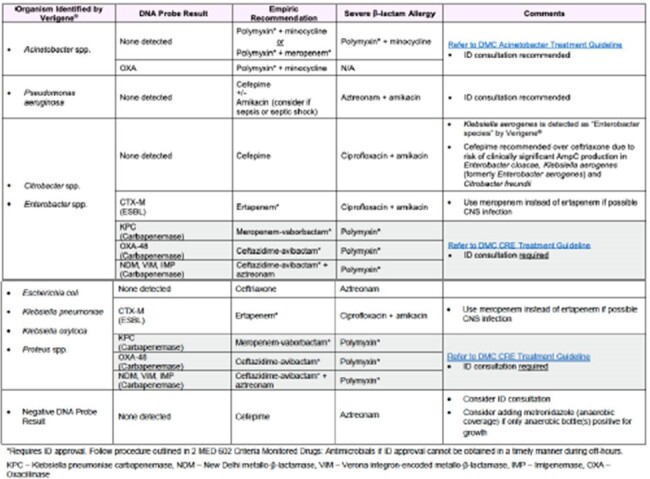
Institutional Gram-negative therapy protocol.

**Disclosures:**

**Kaylee E. Caniff, PharmD, BCIDP**, T2Biosystems: Honoraria **Michael J. Rybak, PharmD, PhD, MPH**, Abbvie, Melinta, Sionogi, Merck, T2Biosystems: Advisor/Consultant|Abbvie, Melinta, Sionogi, Merck, T2Biosystems: Grant/Research Support|Abbvie, Melinta, Sionogi, Merck, T2Biosystems: Speaker

